# The Mechanism of the Gut-Brain Axis in Regulating Food Intake

**DOI:** 10.3390/nu15173728

**Published:** 2023-08-25

**Authors:** Shouren Li, Mengqi Liu, Shixi Cao, Boshuai Liu, Defeng Li, Zhichang Wang, Hao Sun, Yalei Cui, Yinghua Shi

**Affiliations:** 1 College of Animal Science and Technology, Henan Agricultural University, Zhengzhou 450002, China; lsraaa577@163.com (S.L.); 2019110376@sdau.edu.cn (M.L.);; 2 Henan Key Laboratory of Innovation and Utilization of Grassland Resources, Zhengzhou 450002, China; 3 Henan Forage Engineering Technology Research Center, Zhengzhou 450002, China

**Keywords:** gut-brain axis, food intake regulation, gut-brain peptides, gut microbes, bacterial metabolites, appetite

## Abstract

With the increasing prevalence of energy metabolism disorders such as diabetes, cardiovascular disease, obesity, and anorexia, the regulation of feeding has become the focus of global attention. The gastrointestinal tract is not only the site of food digestion and absorption but also contains a variety of appetite-regulating signals such as gut-brain peptides, short-chain fatty acids (SCFAs), bile acids (BAs), bacterial proteins, and cellular components produced by gut microbes. While the central nervous system (CNS), as the core of appetite regulation, can receive and integrate these appetite signals and send instructions to downstream effector organs to promote or inhibit the body’s feeding behaviour. This review will focus on the gut-brain axis mechanism of feeding behaviour, discussing how the peripheral appetite signal is sensed by the CNS via the gut-brain axis and the role of the central “first order neural nuclei” in the process of appetite regulation. Here, elucidation of the gut-brain axis mechanism of feeding regulation may provide new strategies for future production practises and the treatment of diseases such as anorexia and obesity.

## 1. Introduction

As one of the basic physiological activities of humans, feeding is essential for maintaining the body’s vital activities and energy homeostasis. If the organism’s long-term energy intake is less than its energy expenditure, a number of dysfunctions can be triggered, which in severe cases can be life-threatening. The regulation of body feeding is influenced by a variety of factors, including (i) Food quality, freshness, and composition, which affect intake; even the simplest salt requirement can affect feeding by influencing food salinity. (ii) External environmental pressures: approximately 35 to 60 percent of people reported that stress increased their food intake, while 25 to 40 percent reported that stress decreased their food intake, depending on the type of stress they were under [1]. (iii) The physiological state of the organism, such as the late gestation period of the mother, where the uterine contents compress the gastrointestinal tract, causing contraction of the gastrointestinal tract, resulting in a decrease in feeding and a significant increase in food intake after delivery. Previous studies may have focused more on the effects of external factors on foraging and lacked an in-depth investigation of the mechanisms.

For the past few years, the gut-brain axis involved in food intake regulation has increasingly become a leading research issue, and great progress has been made at the genetic, metabolic, and neural pathway levels involved in the regulation of food intake. The intestinal tract is able to fully perceive the nutritional status of the organism after ingestion and transmit it via the gut-brain axis to the brain, which in turn regulates food intake and maintains the body’s energy metabolism [2]. As key appetite signalling molecules, gut-brain peptides, gut microbes, and their metabolites play an important role in this process [3]. This review explains the role of “first-order neural nuclei” in the brain’s feeding regulatory circuit and details how gut-brain peptides, gut microbes, and their metabolites control the organism’s feeding behaviour.

Compared to previous articles that focused solely on the effects of gut-brain peptides on food intake [4], this article provides a more comprehensive review of the pathways involved in the regulation of feeding behaviour through microbe-gut-brain interactions. We believe that this analysis will lay the foundation not only for future research aimed at exploring new mechanisms of appetite regulation by the gut-brain axis but also for future livestock production practices.

## 2. The Role of “First-Order Neural Nuclei” in Food Intake Regulation

The gut-brain axis is a bidirectional communication system formed by the CNS and the gastrointestinal tract, involving humoral pathways such as gut-brain peptides, gut microbial metabolites, and cytokines, and neural pathways such as the vagus nerve, spinal nerve, and autonomic nervous system, which can condition many physiological functions such as behavioural regulation and immune response [5]. As the starting point of the brain’s feeding regulation circuit, the “first-order neural nuclei“ play an indispensable role in energy homeostasis mediated by the gut-brain axis.

### 2.1. Arcuate Nucleus (ARC)

The ARC of the hypothalamus, as one of the most important nuclei for sensing the energy levels of the organism, contains two peptide-producing neurons responsible for appetite regulation: The neuropeptide Y/agouti-related peptide (NPY/AgRP) neurons and the pro-opiomelanocortin (POMC) neurons [6]. Because of the abundant blood supply and the weak blood-brain barrier in the third ventricle, NPY/AgRP neurons and POMC neurons can directly sense a variety of signalling molecules in body fluids involved in appetite regulation; hence, the ARC is often considered the ‘first-order neural nuclei’ in humoral regulatory pathways [7].The ARC integrates appetite-regulating signalling molecules from the circulation and then sends hunger or satiety signals via neural projections to other nuclei within the CNS, which together form a complex feeding regulatory circuit that in turn stimulates or inhibits feeding behaviour in organisms [8].

In addition to producing AgRP, 90% of AgRP neurons co-express the neuropeptide NPY, which is why they are also called NPY/AgRP neurons. Activation of AgRP neurons promotes feeding behaviour even under conditions of appetite suppression and decreases neural activity in the anorexigenic Parabrachial nucleus (PBN) [9]. Dopamine receptor D1 (Drd1) is also expressed on AgRP neurons, and upregulation of Drd1 activity induces the ingestion of high-fat and high-sugar foods [10]. Dynamin-related protein1 (Drpl), a key protein, mediates mitochondrial fission and fatty acid oxidation in AgRP neurons as an important mechanism for AgRP neurons to promote food intake [11]. Killing AgRP neurons in adult mice using diphtheria toxin by Luquet et al. resulted in decreased appetite, whereas knocking out these neurons in young mice did not affect feeding [12], elaborating that a compensatory mechanism is formed in young mice to maintain food intake.

POMC proteins act as precursors for a wide range of functional peptides [13], which are converted to α-Melanocyte-stimulating hormone (α-MSH), β-MSH, γ-MSH, β-endorphin and Adrenocorticotropic hormone (ACTH) by the cleavage of Prohormone convertase 2 (PC2) and 1/3 (PC1/3) [14]. Among these, MSH acts as an important activating ligand for melanocortin receptor-4 (MC4R) and MC3R, and activated MC3/4R can reduce appetite and increase energy expenditure [15]. In contrast, during energy deprivation, AgRP peptides secreted by AgRP neurons act as inverse agonists of MC4R, reducing MC4R activity and promoting feeding behaviour [16]. It is widely accepted in the field that POMC neurons reduce appetite and induce satiety; however, it is noteworthy that cannabinoid-activated POMC neurons promote ingestion by inducing the release of appetite-stimulating β-endorphin rather than α-MSH [17].

**Figure 1:**
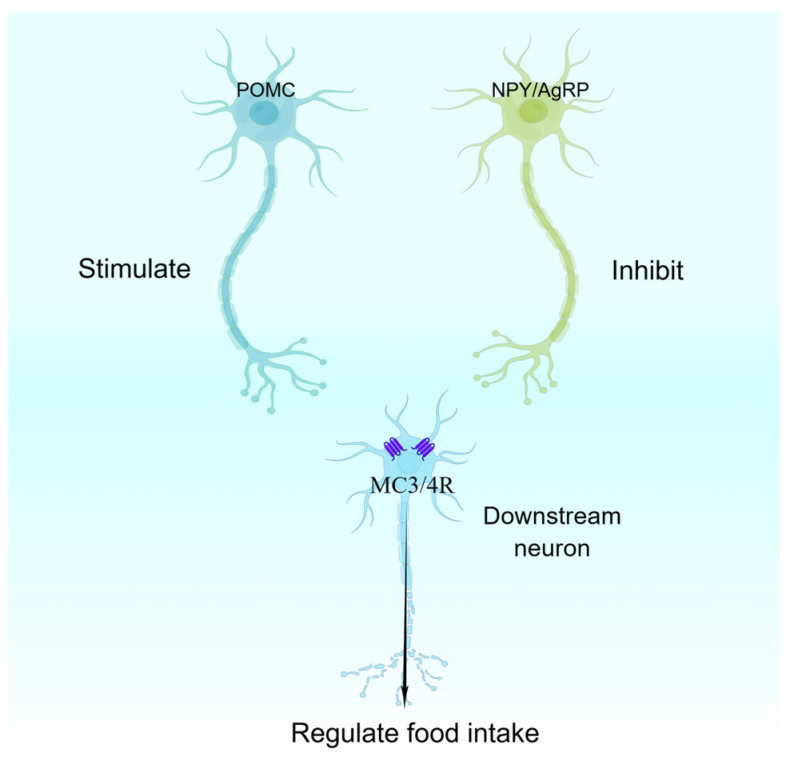
**Central melanocortin system.** AgRP, agouti-related peptide; NPY, neuropeptide Y; MC3/4R, melanocortin receptor-3/4; POMC, pro-opiomelanocortin.

POMC neurons, NPY/AgRP neurons, and downstream neurons expressing MC3/4R together form the well-known central melanocortin system, which plays an important role in the maintenance of organismal energy homeostasis [16] (Figure 1).

### 2.2. Nucleus Tractus Solitarii (NTS)

The vagus nerve is an important link between the peripheral organs and the brainstem NTS, connecting the gut and other organs at one end, sensing the energy state of the organism, and the NTS at the other end, integrating nutritional signals from the gastrointestinal tract and thus regulating feeding behaviour and energy metabolism [18]. The gastrointestinal system releases nutritional signals such as glucagon-like peptide-1 (GLP-1), Ghrelin, and cholecystokinin (CCK), depending on the nutritional status of the body, while the vagal afferent nerve terminals express GLP-1 receptors, Ghrelin receptors, and CCK receptors, and the appropriate nutrient signals bind to these receptors, which are then transmitted to the brain via the vagus nerve to control appetite [19], so the NTS is often referred to as the “first-order nuclei” of the vagal afferent nerve to the CNS. Due to research innovations, researchers discovered neuropod cells in the intestinal wall, which form synaptic connections with the vagus nerve and transfer with the help of glutamate as a neurotransmitter, allowing the brain to respond more quickly and accurately to intestinal signals [20,21]. In addition to vagal connections, the NTS also directly senses nutrient molecules in the humoral circulation as well as neural signals projected from the forebrain, which integrate multiple energy state signals and transfer them to other nuclei to jointly control energy metabolism [22]. Upregulation of calcitonin receptor neuron activity in the NTS was found to induce non-aversive feeding inhibition [23]; stimulation of NTS POMC neurons also rapidly enhanced organ satiety [24]; chemical activation of NTS A2 neurons via projections to the paraventricular nucleus of the hypothalamus (PVN); however, not the bed nucleus of the stria terminalis (BNST), reduced food intake [25]. Overall, it is generally accepted that the vagal-brain neural circuit mediates postprandial satiety signals, which cause feeding behaviour to cease [26]. However, Chen et al. recently found that upregulation of the activity of neurons co-expressing NPY and catecholamines in the NTS occurs in response to hunger signals that elicit food intake [27].

**Figure 2:**
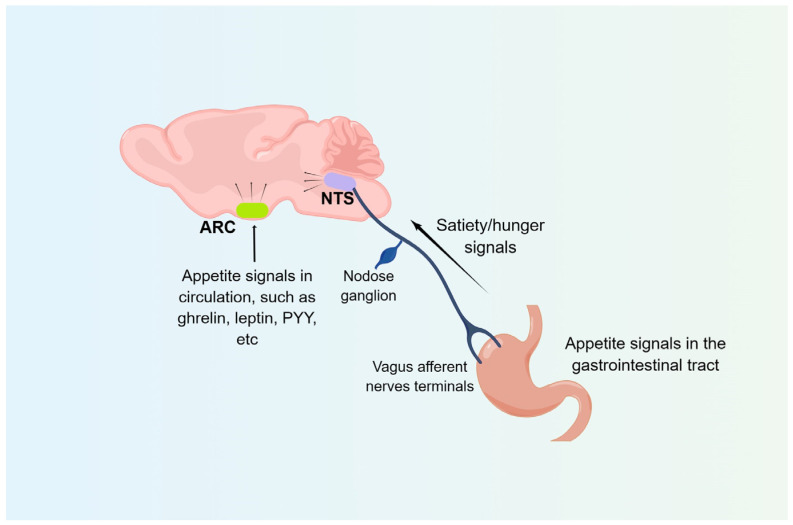
**Food intake regulatory pathways.** The ARC receives humoral signals, while the NTS mainly receives signals from the gastrointestinal tract via the vagus afferent nerves. ARC, arcuate nucleus; NTS, nucleus tractus solitarii; PYY, peptide YY.

In summary, the ARC and NTS, the two “first-order neural nuclei” that are the starting point of the appetite control loop in the brain, can respond to satiety/hunger signals from the gastrointestinal tract to regulate the body’s food intake and energy balance (Figure 2). 

## 3. The Effect of Typical Gut-Brain Peptides on Food Intake Regulation

**Figure 3:**
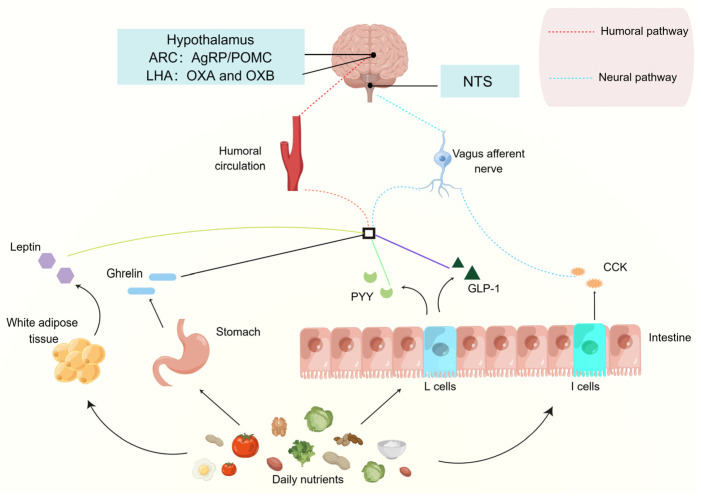
**Diagram of Gut-brain peptides regulate appetite.** The nutrients in the diet can stimulate the corresponding tissues and organs to secrete gut-brain peptides into the circulation and then transmit appetite signals to the brain through neural and humoral pathways, mediating the feeding behaviour of the organism. AgRP, agouti-related peptide; ARC, arcuate nucleus; CCK, cholecystokinin; GLP-1, glucagon-like peptide-1; LHA, lateral hypothalamic area; NPY, neuropeptide Y; NTS, nucleus tractus solitarii; OXA, orexin A; OXB, orexin B; PYY, peptide YY; POMC, pro-opiomelanocortin.

In the gut-brain axis, the gastrointestinal tract plays an essential role, directly contacting and digesting food, sensing changes in the body’s nutritional needs, and releasing gut-brain peptides, a signalling molecule that is transferred into the circulation to manipulate the activity of the associated appetite neurons, thereby maintaining the energy homeostasis of the organism (Figure 3).

### 3.1. Orexin

Orexin A (OXA) and OXB, a pro-feeding gut-brain peptide, are formed by enzymatic cleavage of the precursor molecule prepro-orexin (PPO) and secreted mainly from the lateral hypothalamic area (LHA) [28]. Molecular and immunohistochemical techniques have demonstrated that the orexin receptor (OXR) is widely distributed throughout the brain, with the most abundant expression in the hypothalamus [29]. OXR is a member of the G protein-coupled receptor (GPR) family and has two receptor subtypes, OX1R and OX2R. OX1R has a higher affinity for OXA, while OX2R has a similar affinity for both [28]. OXR binds to its corresponding ligand and initiates intracellular signalling pathways that regulate feeding, neuroendocrine, and sleep/wake behaviour in mammals [30]. Experiments have shown that ventricular injections of both OXA and OXB stimulate food intake; however, OXA has a significantly greater feeding effect than OXB [31], a process that is blocked by OXR antagonists, inducing satiety and reducing body weight [32]. Microinjection of exogenous OXA into the Central amygdala (Cea) increases the intake of high-fat foods without affecting the normal food intake; however, this effect is partially blocked by Drd1 antagonists [33], suggesting that OXA (Cea) is also involved in the composition of the hedonic feeding loop. Endogenous androgens, possibly testosterone, reduce glucose-deprivation-induced feeding behaviour in animals by down-regulating the activity of OXA neurons [34]. The researchers found that orexin-induced feeding behaviour is mainly mediated by the NPY system in the ARC and that the use of NPY receptor antagonists to some degree reversed the feeding-promoting effects of Orexin [29]. Furthermore, a study by Morello et al. showed that there is a significant negative correlation between OXA and α-MSH in obese mice due to OXA binding to the OX1R on POMC neurons and inhibiting *POMC* gene transcription through a series of signalling events, which in turn reduces α-MSH production [35]. The central function of Orexin has been extensively studied; however, its role in peripheral tissues such as the gut, fat, and liver are becoming a source of discoveries and new research. In the future, a deeper understanding of the mechanism of action of orexin and its downstream effects will bring different insights into improving the health and nutrition of the organism.

### 3.2. Ghrelin

Ghrelin, also known as growth hormone secretagogue peptide, is produced by X/A-type (rodents) and P/D1-type (humans) digestive tract mucosal cells [36] and plays a wide range of roles in food intake control, gastrointestinal inflammation, and cardiovascular regulation [37]. The *Ghrelin* gene encodes for the production of Ghrelin precursor proteinogen (Preproghrelin), consisting of 117 amino acids, and this peptide produces different subproducts in response to a series of enzymes, of which acylated Ghrelin (Acyl-ghrelin, AG) and deacylated Ghrelin (Des- acyl-ghrelin, DAG) are the most abundant, both consisting of 28 amino acids [38]. Ghrelin’s N-terminal serine-3 (Ser-3) is acylated with n-octanoic acid under the mediation of Ghrelin O-acyltransferase (GOAT), a modification that is essential for Ghrelin to recognise and activate relevant receptors [39]. The Ghrelin receptor (Growth hormone secretagogue receptor, GHSR), a member of the GPR family, can heterodimerize with other weight-regulating GPRs such as MC3R, Drd1, and OX1R, which interact to jointly regulate the energy homeostasis of the organism [40].

Under normal physiological conditions, ghrelin levels in the body increase gradually during fasting, peaking before feeding and decreasing rapidly after feeding, and there is considerable evidence that this change is closely related to circulating nutrients [41], such as glucose, amino acids, fatty acids, and trace elements. Studies have shown that Ghrelin has the ability to promote food intake, increase body weight, and accelerate energy expenditure in animals. Mice injected subcutaneously with the GHSR agonist JMV 1843 for 10 days showed a significant increase in food intake and a significant increase in body weight compared to mice injected with saline [42]. Feeding tryptophan to weanling pigs resulted in increased plasma Ghrelin levels as well as significantly enhanced feeding activity at 2 h, 8 h, and 24 h [43]. It was found that Ghrelin’s appetite-stimulating mechanism is achieved mainly through two feeding regulatory pathways: humoral and neural [7]. Secreted ghrelin reaches the ARC via the humoral circulation and activates the GHSR on NPY/AgRP neurons, significantly increasing the activity of these neurons and inhibiting the activity of anorexigenic neurons, thereby increasing food intake and body weight [44]. Notably, however, GHSR was not expressed on POMC neurons, suggesting that inhibition of POMC neurons is achieved indirectly via the inhibitory neurotransmitter γ-aminobutyric acid (GABA) released by NPY/AgRP neurons [45]. Ghrelin also binds to the GHSR on vagal afferent nerves, transmitting hunger signals to brain regions involved in energy regulation and stimulating feeding behaviour in animals [7]. In addition, Bruschetta et al. reported that Ghrelin reduces hypothalamic α-MSH levels by stimulating AgRP neurons to secrete prolyl carboxypeptidase (PCRP), which in turn produces a stronger appetitive drive [46].

### 3.3. CCK

CCK is mainly secreted by type I cells of the small intestinal mucosa in amounts proportional to dietary protein and lipid levels and is widely distributed in the gastrointestinal tract and the central and peripheral nervous systems. Researches has shown that CCK acts as a satiety signal and can perform a variety of biological functions, including stimulating pancreatic secretion, contracting the gallbladder, and delaying gastric emptying [47]. CCK exists in a variety of active molecules due to differences in translation and processing, with CCK-58, CCK-8, and CCK-33 being the major molecular forms [48]. The presence of two CCK receptor subtypes, the CCK-1 receptor (CCK-1R) and the CCK-2R, can be observed in the organism. In rats, CCK-1R is distributed in the pancreas, gallbladder, vagal afferent nerve, and certain areas of the brain and is mainly involved in the regulation of feeding; CCK-2R is expressed in the gastric mucosa, CNS, and vagus nerve [49]. In a study by L. Wang et al., dietary soy protein may trigger the secretion of CCK through activation of the calcium-sensing receptor (CaSR) and intracellular Ca^2+^/TRPM5 pathway, resulting in a decrease in appetite [50]. A more rapid release of CCK was observed when spinach extract was given orally to rats, which in turn induced an earlier sensation of satiety, an effect that may arise from the higher content of flavonoids in spinach [51]. As well, intraperitoneal injection of CCK in blind cavefish, *Astyanax fasciatus mexicanus*, significantly reduced feed intake compared to the saline-injected group [52].

In previous reports, peripheral CCK-mediated decreases in feeding activity were mainly mediated via the vagal pathway [53]. Decreasing vagal sensitivity to CCK in rats can cause dysregulation of food intake [54]. One hour after peripheral injection of CCK-8 in goldfish, the researchers found significantly higher levels of *POMC* mRNA in brain tissue, indicating that peripheral CCK signalling mediates appetite loss via vagal afferent neurotransmission to the hindbrain and subsequently through the POMC signalling pathway [55]. Fan et al. also demonstrated that POMC neurons are also expressed in the caudal part of the NTS and can be activated by electrical or CCK-induced stimulation of the vagal afferent nerve [56]. In addition, optogenetic activation of CCK-expressing neurons in the NTS, which send axonal projections to the PVN, induces satiety and reduces body weight in mice [57]. In short, CCK can be used as a general gastrointestinal hormone to regulate gastrointestinal motility, and it can also act as a neurotransmitter in the CNS. At present, there are many studies on the mechanism of CCK in regulating animal feeding but fewer reports on the application of CCK in production practice, so it is undoubtedly of great practical importance to intensify research in this area.

### 3.4. GLP-1

GLP-1, a product encoded by the *proglucagon* gene, is mainly synthesised and secreted by distal L-cells of the small intestine. Research has reported that GLP-1 is also produced in the brain by preproglucagon (PPG) neurons in the NTS and that this region serves as the major source of central endogenous GLP-1 [58]. In mouse models, GLP-1 receptor (GLP-1R) expression is observed in both the peripheral (pancreas, gastrointestinal tract, kidney, etc.) and CNS (hypothalamus, hippocampus, brainstem, etc.) [59]. It has been reported that active GLP-1 secreted from the small intestine crosses the blood-brain barrier but is rapidly degraded to inactive fragments in the circulation [60], demonstrating that only a minority of peripherally active GLP-1 reaches the CNS. The researchers then measured GLP-1 levels in brain tissue and found that GLP-1 levels in the brain were many times higher than in the circulation [58]. These examples all support the view that centrally active GLP-1 is predominantly derived from NTS PPG neurons and not from the peripheral circulation.

Intracerebroventricular injection of Exendin-4 (Ex-4), a GLP-1R-specific agonist, significantly reduced food intake and decreased body weight as well as hypothalamic and gastric ghrelin levels in rats [61], consistent with Hong et al., finding that Ex-4 inhibits Ghrelin secretion via the Mammalian target of Rapamycin (mTOR) signaling pathway [62]. GLP-1 analogues were found to increase POMC neurons’ activity in a time-dependent manner after intraperitoneal and subcutaneous injections in mice [63]. Moreover, NTS PPG neurons can send dense projections to multiple regions of the brain, e.g., dense mapping to the ARC region increases the frequency of action potential discharges of POMC neurons [64]; reverse tracing and immunohistochemical techniques have shown that inputs from NTS PPG neurons to the PVN mediate the generation of satiety in the organism [65]; and that central GLP-1 reduces the excitability of dopamine neurons in the ventral tegmental area (VTA) of the midbrain and decreases the intake of high-fat foods by inhibiting the hedonic pathway [66]. In conclusion, GLP-1R-expressing neuronal nuclei in the brain respond to both NTS PPG neurons and GLP-1 signals from the gastrointestinal tract to induce a feeling of satiety in the body. 

### 3.5. Peptide YY (PYY)

PYY is an anorexigenic brain-gut peptide that is released in response to food intake, primarily by L-cells in the small intestine, and is distributed in sequentially increasing concentrations from the foregut to the hindgut [67]. Keire et al. found that PYY_1–36_, as the major form secreted from the distal small intestine of rats, is enzymatically converted in the circulation by dipeptidyl peptidase-4 (DPP4) to the more active molecular form PYY_3–36_ [68], which induces an enhanced feeling of satiety after a meal. However, when the C-terminus of PYY_3–36_ is removed, PYY-induced anorexia also disappears [69], demonstrating that the integrity of the C-terminus may be essential for PYY to maintain its biological activity. Five GPR isoforms of PPY have been identified, of which the Y2 receptor (Y2-R) has a higher affinity for PYY_3–36_ [70]. Adrian et al. observed low basal levels of PYY in the circulation; however, with the onset of feeding, its secretion is consistent with the nutrients in the diet and remains high for several hours after meal times [71].

Trials have revealed that oral administration of L-arginine upregulates the concentration of PYY in the postprandial circulation and reduces appetite as well as lipid intake [72]. Administration of PYY_3–36_ to mice significantly diminished food consumption, and the effect of injection at night is more obvious than during the day [73]. Co-administration of PYY_3–36_ with Ex-4 subcutaneously resulted in a significant increase in the number of c-fos-positive neurons in the brain compared to when the drugs were administered separately or alone [74], implicating that PYY and GLP-1 may modulate food intake in some synergistic manner [75]. Immunohistochemistry and in situ hybridization have revealed that hypothalamic *Y2-R* mRNA is abundant in NPY-expressing neurons [76], which can be activated by NNC0165-1273 (an analogue of PYY_3–36_), inducing a decrease in NPY/AgRP neurons activity and causing organismic satiety [77], an effect that is resisted when Y2-R is knocked out [78]. PYY_3–36_ also upregulates POMC neuronal activity by decreasing the activity level of NPY nerve terminals [79]. In addition, the vagus nerve partially mediates the satiety signalling of PYY, and PYY_3–36_ induces reduced appetite in rats by stimulating Y2-R on the vagal afferent nerve [80], and the anorexigenic effect of PYY is subsequently attenuated when the bilateral subphrenic vagus nerve is ablated [81].

### 3.6. Leptin

Leptin is an anorexigenic gut-brain peptide encoded by the *ob* gene and secreted mainly in white adipose tissue. The diverse distribution of the leptin receptor (LepR) allows leptin to exert pleiotropic effects, including regulation of food intake, pro-inflammatory immune response, cognition, reproduction, and many other functions [82]. LepRb, a subtype of the receptor, is abundantly expressed in brain regions associated with energy regulation [83] and causes a down-regulation of appetite in the organism. Evidence shows that POMC neurons and NPY/AgRP neurons in the ARC are one of the main targets of Leptin in the brain and that Leptin reduces food intake and increases the body’s energy expenditure by depolarising POMC neurons and hyperpolarising NPY/AgRP neurons [84]. Researchers have further investigated the signalling mechanism of Leptin and found that when Leptin binds to its corresponding receptor, it sequentially phosphorylates and activates Janus-activated kinase 2 (JAK2) and Signal transducer and activator of transcription 3 (STAT3), then pSTAT3 dissociates from LepR and enters the nucleus to bind to *POMC* and *AgRP* genes, promoting transcription of *POMC* mRNA and reducing levels of *AgRP* mRNA [85]. In addition, the LepRs are also expressed on the vagus nerve, and when these receptors are knocked out, increased food intake and weight are observed in animals [86].

Weight gain due to Leptin deficiency can be treated by exogenous injections of Leptin. However, in many people with obesity, Leptin levels are abnormally high compared to normal levels, and this has been attributed to the development of “leptin resistance” [87], i.e., impairment of the Leptin signalling pathway [88]. Moreover, Leptin is potentially associated with gastrointestinal hormones such as Grehlin, CCK, and GLP-1, which collectively exhibit synergistic or opposing appetite-regulating effects [89]; however, the complex feeding mechanisms involved need to be further investigated.

## 4. The Influence of Gut Microbes and Their Metabolites in Food Intake Regulation

**Figure 4:**
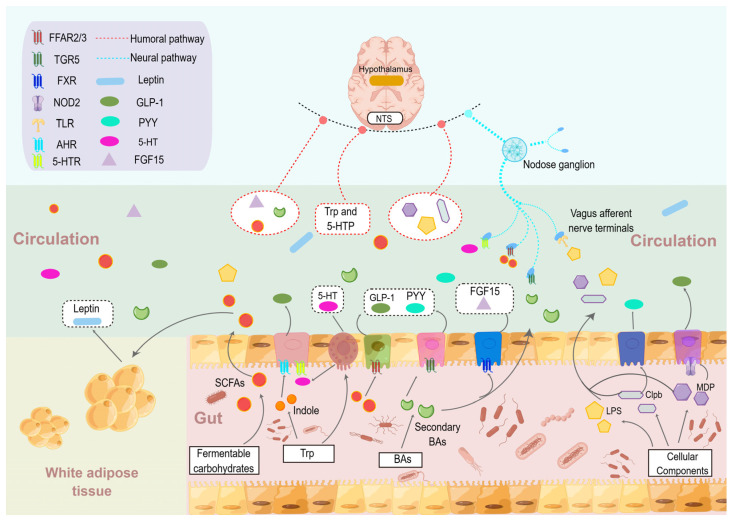
**Gut microbe-brain communication in the regulation of food intake.** Gut microbes rely mainly on their functional metabolites and cellular components to mediate host food intake through humoral, enterodocrine, and neural signalling pathways. AHR, aromatic hydrocarbon receptor; Clpb, caseinolytic peptidase B; FGF15, fibroblast growth factors 15; FXR, farnesoid X receptor; FFAR2/3, free fatty acid receptor 2/3; GLP-1, glucagon-like peptide-1; LPS, lipopolysaccharide; MDP, muramyl dipeptide; NOD2, nucleotide-binding oligomerization domain containing 2; NTS, nucleus tractus solitarii; PYY, peptide YY; SCFAs, short-chain fattyacids; TGR5, takeda G-protein-coupled receptors 5; TLR, Toll-like receptor; Trp, tryptophan; 5-HT, 5-hydroxy tryptamine; 5-HPT, 5-hydroxytryptophan; 5-HTR, 5-HT receptor.

As the largest and most complex micro-ecosystem in the human body, gut microbes play a variety of important roles in the host. There is increasing evidence that gut microbes can communicate with the brain and play a key function in regulating the host’s feeding behaviour and energy homeostasis [90], with functional metabolites such as Indole, SCFAs, and BAs, as well as cellular components and bacterial proteins, serving as intermediate messengers to mediate the communication between the two (Figure 4).

### 4.1. SCFAs

Researchers have found that adding dietary fibre to meals could suppress appetite and improve body weight [91,92], and these changes were subsequently confirmed to be associated with SCFAs, a fermentation product of the colon [93]. In the study by Brown et al., although all three major SCFAs (acetic, propionic, and butyric) activated the free fatty acid receptor 2 (FFAR2)/GPR43 and FFAR3/GPR41, FFAR2 had a higher affinity for acetic acid, whereas propionic and butyric acids were potent FFAR3 agonists [94]. Both receptors are expressed in enteroendocrine L cells, while FFAR3 is also observed in the peripheral nervous system [95].

The available evidence suggests that SCFAs maintain energy homeostasis in the body either by entering the brain via the somatic circulation, stimulating the secretion of gut-brain peptides, or mediating the afferent transmission of appetite signals via the vagus nerve [96]. PET-CT scans of mice injected intraperitoneally with 11C-acetate solution show that acetate crossed the blood-brain barrier to reach the ARC region and induced elevated neuronal activity in POMC as well as decreased AgRP neuronal activity [97]. At the same time, acetate also stimulates Leptin secretion from the white adipose tissue through a FFAR2-dependent mechanism [98], causing changes in the activity of appetite-related neurons in the brain. Psichas et al., injected propionate into the colon, which caused activation of FFAR2 on intestinal L cells, and observed elevated levels of PYY and GLP-1 in plasma from the rat jugular vein and mouse portal vein [99]. In research by Larraufie et al., butyric acid, the most potent agonist of anorexigenic peptides in SCFAs, increased the expression of the *PYY* gene 120-fold, whereas propionic acid and acetic acid increased it by 40-fold and 2-fold, respectively [100]. Oral (not intravenous) butyric acid significantly reduced the excitability of hypothalamic NPY-expressing neurons and the number of c-fos-positive neurons in the NTS and dorsal vagal complex (DVC); however, had no effect on the activity of POMC neurons [101]. In addition, the vagal afferent nerves are an important aspect of appetite regulation, and the researchers found that knocking out FFAR3 on the terminals of the vagal afferent nerve led to an increase in food intake in mice [102]. Failure of butyric acid-treated groups to reduce cumulative food intake in comparison with controls after subdiaphragmatic vagus nerve transection in mice [101], implying that the vagus nerve is essential for butyrate-induced satiety in the organism. At present, there is no doubt that SCFAs act as a satiety signal in vivo; however, the signal transduction mechanisms mediated by them are still at the primary research stage, and exploring the detailed mechanisms of SCFAs will help us better understand their effects on appetite regulation.

### 4.2. BAs

Elementary BAs are converted from cholesterol in the liver, released into the intestinal lumen during the digestion of chyme by the organism, and converted to secondary BAs such as deoxycholic acid (DCA) and lithocholic acid (LCA) by uncoupling and dehydroxylation in the presence of bile salt hydrolase-expressing intestinal microorganisms such as *Clostridium* spp., *Lactobacillus* spp., *Enterococcus* spp., and so on [103]. Recent research has revealed that in addition to affecting lipid metabolism and inflammatory response, BAs also play a role in regulating appetite and energy homeostasis [104], where farnesoid X receptor (FXR) and takeda G-protein-coupled receptor 5 (TGR5) act as crucial receptors to mediate BAs’ function.

The research demonstrated that BAs are potent agonists that promote the release of GLP-1 and PYY, which upregulate the activity of TGR5 in intestinal L-cells, significantly increasing colonic levels of GLP-1 (a 3.5-fold increase compared to control) and PYY (a 2.9-fold increase compared to control) [105]. While knocking out TGR5, small intestinal perfusion with BAs did not induce secretion of the anorexigenic peptides GLP-1 and PPY [106]. Meanwhile, BAs also triggers FXR in the distal small intestine to secrete fibroblast growth factor (FGF) 15 into the circulation and cross the blood-brain barrier to interact with AgRP/NPY neuronal FGF receptors, subsequently leading to a series of signal transduction events to diminish *AgRP/NPY* gene expression [107], which indicates that the BAs-FXR-FGF15 pathway is an instrumental part of maintaining energy homeostasis in the organisation. Additionally, the presence of BAs in the brain was also observed by Higashi et al. and was positively correlated with the level of BAs in the plasma [108], leading to the hypothesis that BAs in the peripheral circulation could cross the blood-brain barrier into the brain. Using RNA Scope brain expression profiling, *TGR5* mRNA was found to be highly expressed in the ARC area of the hypothalamus, and oral or intravenous administration of the TGR5 agonist INT-777 to mice abolished feeding behaviour by inhibiting *AgRP/NPY* mRNA expression and neuropeptide release [109]. Furthermore, the vagus nerve is also involved in BAs mediating satiety, and TGR5 on it transduces afferent DCA anorexic signals that specifically trigger the activation of POMC anorexic neurons in the hypothalamus, causing a reduction in food intake [110].

### 4.3. Tryptophan-Derived Metabolites

Tryptophan (Trp) is an essential amino acid and the only one with an indole structure, mainly provided by dietary protein. Agus et al. reported that Trp in the gut, under the direct or indirect regulation of the flora, can generate various active molecules mainly through three metabolic pathways (the serotonin pathway, the indole pathway, and the kynurenine pathway), which in turn mediate the body’s metabolism, immunity, and gastrointestinal function [111].

Tryptophan hydroxylase (TPH) serves as the key rate-limiting enzyme in the serotonin pathway, with two isoforms, TPH1 (mainly found in enterochromaffin cells, ECs) and TPH2 (mainly found in the brain), in which the enzyme hydroxylates Trp to produce 5-hydroxytryptophan (5-HTP), which is then converted through a series of metabolic pathways to serotonin (5-hydroxy tryptamine, 5-HT) [112]. Meanwhile, gut microbes are also major participants in regulating 5-HT production [113], e.g., SCFAs produced by the flora increase the expression level of ECs *TPH1* mRNA, which in turn accelerates the biosynthesis of 5-HT [114], and other studies have demonstrated that spore-forming microbes from mice and humans can also promote 5-HT production [115]. The fact that 5-HT, whether of gut or brain origin, induces satiety in the organism has long been undisputed [116]. In vitro assays showed that 5-HT (30 μM or 100 μM) significantly upregulated the ability of the mouse small intestinal endocrine cell line STC-1 to release GPL-1 compared to control, whereas it did not induce the above changes when a non-specific 5-HT receptor antagonist was used [117], suggesting that there may be a reciprocal mechanism by which 5-HT may be involved in the secretion of GLP-1. Although gut-derived 5-HT does not cross the blood-brain barrier, its precursors, Try and 5-HTP, do, indirectly influencing central 5-HT function and production [118]. Existing studies have shown that the ARC, PVN, and neuronal nuclei expressing 5-HT1A receptor(5-HT1AR), 5-HT1BR, 5-HT2AR, and 5-HT2CR are the main targets of 5-HT in the brain, mediating the feeding behaviour of the organism. Activation of 5-HT1BR significantly inhibits the ingestive effects of NPY/AgRP neurons and increases the feeling of satiety in the body [119]. In total, 5-HT also binds to 5-HT2CR on anorexigenic neurons, POMC, upregulating the level of central α-MSH biosynthesis [120]. In addition, the vagus nerve acts as a sensory transducer that transmits 5-HT signals from the gut to the brain [121]. The diversity of 5-HT receptors and their widespread distribution mean that 5-HT has a wide range of biological functions. Current research points to a number of 5-HT receptors being involved in the regulation of feeding, but which receptors play a major role and how they work together need to be further confirmed.

Previous research has shown that up to 85 strains can encode *tryptophanase* genes, and tryptophan can be fermentatively produced in these colonies to form indole, a ligand for aromatic hydrocarbon receptors (AHR) [122]. Enhanced GLP-1 secretion was observed after stimulation of the mouse GLUTag cell line using Ficz, an AHR agonist [123]. This is consistent with the results of Chimerel et al., that indole induces a rapid release of GLP-1 from intestinal L-cells in the short term but inhibits its secretion in the long term, a phenomenon that appears to be related to the production and use of adenosine triphosphate in the cells [124]. The above results indicate that indole may mediate host appetite by affecting the secretion of gut-brain peptides. In addition, indole and its derivatives may affect the balance of human gut microbes by inhibiting fungal growth and modulating flora motility, colonisation and biofilm formation [122]; however, it is not clear whether this flora balance is relevant to host appetite regulation.

### 4.4. Bacterial Proteins and Cellular Components of Gut Microbe

Further research has shown that gut microbes produce bacterial proteins that affect brain areas involved in energy regulation, as caseinolytic peptidase B (Clpb) protein homologs act as conformational antigen-mimetic of α-MSH [125], i.e., Clpb has homology with the amino acid sequence of α-MSH. Clpb protein homologs can be produced by flora, which includes *E. coli* [126,127]. Measurement of Clpb levels in healthy and anorexic populations using immunoassays showed that Clpb levels were higher in anorexic patients and did not differ significantly between patient subgroups [128], and reduced levels of anti-Clpb immunoglobulin M (IgM) and IgG were observed in patient plasma, so it was hypothesised that the enhanced satiety sensation mediated by Clpb may result from decreased levels of humoral immunity [129]. Macrogene sequencing has shown that the relative abundance of the Clpb-producing phylum and family is decreased in obesity patients [130]. Treatment of *ob/ob* mice with *Hafnia alvei*, a potential probiotic that secretes Clpb protein, significantly decreased food intake as well as weight and fat gain [131,132]; however, did not produce the above changes when given to a Clpb-deficient strain [133]. Manon Dominique et al. added equal calories of bovine serum proteins, D-fructose, and oleic acid to *E. coli* cultures and found that the supplementary protein group upregulated bacterial *Clpb* mRNA expression [134], suggesting that protein-mediated satiety may be related to bacterial secretion of Clpb. The researchers then explored the mechanism of Clpb-induced anorexia and found that Clpb increased PYY secretion in primary cultured cells of rat intestinal mucosa in a dose-dependent manner [134,135], demonstrating that Clpb activates the appetite signalling pathway mediated by PYY to suppress food intake. Moreover, the presence of Clpb in the hypothalamus of rodents and humans can be observed using protein immunoblotting [136]. Clpb injected intraperitoneally reaches the hypothalamus via the systemic circulation and increases the number of c-fos-positive neurons in anorexic neurons, with repeated injections causing a reduction in food intake in mice [137].

In parallel, other trials have demonstrated that Lipopolysaccharide (LPS) and Muramyl dipeptide (MDP) serve as modulators of food intake. As a major component of the outer membrane of Gram-negative bacteria, LPS binds to Toll-like receptors (TLRs) and then undergoes a series of signalling pathways that ultimately trigger metabolic dysfunction and disease development in the body [138]. It was found by Breen et al. that LPS reduced appetite and body weight and increased the incidence of inflammation and mortality in mice [139], whereas injection of agmatine into the brain ventricles normalised LPS-mediated disorders, including anorexia nervosa [140]. In broiler chickens injected intraperitoneally with LPS, a decrease in the levels of *AgRP* mRNA and *NPY* mRNA was clearly observed, while the expression of anorexigenic neuropeptides was unchanged [141], implying that the decrease in the expression levels of *AgRP* and *NPY* genes might be one of the main reasons for the anorexia induced by LPS. Further studies uncovered that blockade of the mTOR signalling pathway with rapamycin attenuated LPS-induced appetite reduction and suppression of *AgRP* gene expression [142], demonstrating that LPS can exert its anorectic effects through the mTOR-AgRP pathway. LPS can also increase the excitability of vagal afferent nerves through a TLR4-dependent mechanism [143], yet cutting subphrenic vagal afferent nerves did not increase food intake [144]. These findings provide support for the idea that LPS can co-regulate the energy metabolism of the organisation through both neural and humoral pathways. As an agonist of the nucleotide-binding oligomerization domain-containing 2 (NOD2) pattern recognition receptor, MDP is considered a marker of bacterial proliferation in the host organism. In previous reports, MDP was shown to significantly increase satiety [145], which was attributed to the presence of NOD2 receptor expression on mouse intestinal L-cells, leading to a significant increase in GPL-1 secretion when treated with MDP derivatives under normoglycaemic conditions [146]. Additionally, Gabanyi et al. observed that certain regions of the brain can be targeted by radioisotope-labelled MDP and that specific knockout of the hypothalamic NOD2 receptor upregulates appetite and body weight in mice [147], confirming that hypothalamic neurons can directly sense changes in gut microbes and adjust the organism’s appetite accordingly.

## 5. Conclusions and Prospect

**Figure 5:**
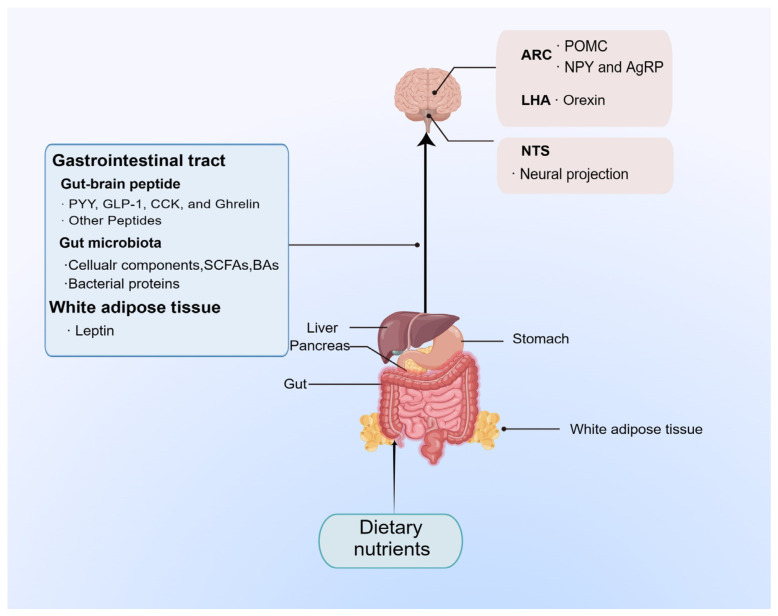
**Diagram of the gut-brain axis that regulates food intake.** Gut-brain peptides, gut microbes, and their metabolites transmit the nutritional state of the body to the brain through the gut-brain axis pathway and thus regulate the food intake of the body by affecting the activity of appetite related neurons. AgRP, agouti-related peptide; ARC, arcuate nucleus; BAs, bile acids; CCK, cholecystokinin; GLP-1, glucagon-like peptide-1; LHA, lateral hypothalamic area; NPY, neuropeptide Y; NTS, nucleus tractus solitarii; PYY, peptide YY; POMC, pro-opiomelanocortin; SCFAs, short-chain fatty acids.

In general, the gut-brain axis serves as an inevitable chain in regulating the food intake of organisms, in which the gut-brain peptides, intestinal microorganisms, and their metabolites perform significant actions (Figure 5). Gut-brain peptides such as Ghrelin, CCK, and Leptin can enter the CNS directly through the humoral pathway to influence the function of appetitive neurons or indirectly via the vagus nerve to regulate the organism’s feeding behaviour. Gut microbes, however, rely mainly on their functional metabolites and cellular components, such as SCFAs, MDP, and BAs, to cross the blood-brain barrier to reach the brain, pass through vagal afferent neural pathways, or stimulate enteroendocrine cells to release the PPY and GPL-1 pathways, which work together to regulate food intake and energy homeostasis in the body.

As technology advances, research into the gut-brain axis involved in appetite regulation is becoming increasingly popular; however, there are three points that deserve particular attention in future research: (i) The majority of existing studies have focused on gut-brain peptides working separately, and there is a lack of exploration to unify them. Trials have indicated that greater anorexic impulses are produced when PYY and GLP-1 are co-administered [75]; in the meantime, Blanco et al., also noted that Ghrelin attenuates the satiety mediated by CCK, PYY, and GLP-1 [148]. Enhancing the study of gut-brain peptide interactions and uncovering the complex mechanisms involved may have unexpected effects on the regulation of appetite. (ii) Bacterial flora is one of the most abundant in the organism, with a mass of about 1–1.5 kg and a number of 10^13^–10^14^, which is about 10 times the number of cells in the human body. Many articles have reported that gut microbes affect the CNS; however, the mechanisms involved are lacking. Future research on flora and appetite will not only simply show whether gut microbes regulate ingestion but, more importantly, analyse the pathways through which they influence appetite regulation. (iii) Fecal microbiota transplantation (FMT) can be used to intervene and adjust the composition of the gut microbiome to regulate the host’s feeding behaviour; however, clinical trials of FMT are still in the exploratory stage and face significant challenges. To begin with, the use of FMT has been associated with toxic side effects and even fatalities [149]; furthermore, the structural composition of the feacal flora is complex, and studies have shown that only specific flora in the donor are functioning, so it is essential to tap into the key flora [150]; and lastly, from a moral, ethical, and psychological standpoint, the use of FMT is not acceptable to the general public. FMT research is an inevitable trend; finding the “potential probiotics” in FMT may be an effective strategy for microbial-based appetite treatment. In conclusion, there is no doubt that a significant role is played by the gut-brain axis in the organism, and further exploration of the gut-brain mechanism will be of great revelation and application value.

## Data Availability

Not applicable.
